# The Prince of Wales's Hospital Fund for London

**Published:** 1900-02-17

**Authors:** 


### THE PRINCE OF WALES'S HOSPITAL FUND FOR LONDON.

The annual meeting of the General Councill was held on Tuesday, February 13th, at Marlborough House, the president (H.R.H. the Prince of Wales) in the chair. Present: The Lord-Lieutenant of London (the Duke of Fife), the Bishop of London, the Chief Rabbi (the Rev. Dr. Adler), Viscount Duncannon, Lord Rowton, Lord Iveagh, Lord Farquhar, the Chairman for the School Board of London (Lord Reay), the Governor of the Bank of England (Mr. S. S. Gladstone), the President of the Royal Society (Lord Lister), Sir Savile Crossley, Bart., Sir Trevor Lawrence, Bart., Sir Henry Burdett, K.C.B., Mr. John Aird, M.P., Mr. Sydney Buxton, M.P., Mr. William Latham, Q.C., Mr. Albert G. Sandeman, Mr. H. C. Smith, the Prime Warden of the Fishmongers' Company (Mr. R. B. Martin), Mr. Julius Wernher, and Mr. J. G. Craggs (hon. secretary).

Sir Savile Crossley read letters of regret from the Bishop of Rochester, the Rev. T. Bowman Stephenson, D.D., Lord Rothschild, the Lord Mayor, and the Right Hon. C. Stuart-Wortley, Q.C., M.P.

Mr. H. C. Smith read the draft report of the Council for 1899 as follows:—

### The Policy of the Fund.

The General Council view with satisfaction the work of the Fund during the past year as shown by the reports of the several committees appended hereto. It will be seen that the general policy adopted at the inauguration of the undertaking has been continued, and while money has not been hoarded unnecessarily, the total distributed has left a balance on the right side. The receipts for 1899 are £48,536 15s. 4d., as against £39,272 3s. 5d. of the previous year. This result the Council consider highly satisfactory under the peculiar circumstances of the year. This is more especially the case, seeing that in view of the exceptional claims upon the public the committee decided not to make the usual autumn appeal. It is, however, a noteworthy fact that of all the sums received not one has been in the form of a legacy, although the hospitals themselves derive a large proportion of their incomes from this source. But now that the Fund is proved to have come to stay, it is hoped that exceptions to this rule may be introduced from outside the ranks of existing subscribers.

### The Year's Collection.

The total sum distributed for 1899 amounted to £42,000. Of this sum £26,250 has been given as annual grants, and the balance as donations. It will be seen that in the majority of cases the grants have been given on certain conditions. The cost of management and collection is about 3¾ per cent., as compared with about 6¼ for 1898. As stated by His Royal Highness the President, improvement is evident from the fact that the receipts are larger and the expenses smaller. There has been no clashing with the work of either the Sunday or Saturday Funds, and the promise given to that effect has been kept. Subscribers may be interested to know that most of the surplus copies of the Jubilee programme printed for the benefit of the Fund have been given to London hospitals, Board schools, workhouse infirmaries, and other institutions. In the report of the Hospitals Distribution Committee it will be noted that a distinction is drawn, in those cases in which grants are withheld, between the ten institutions which though favourably reported on, did not at the end of last year appear to be in urgent need of further help, and the three which were not deemed to be hospitals in the true sense of the word. This distinction is drawn by the advice of the Visiting Committee, and in accordance with requests frequently published in the public press.

### The Influence of the Fund.

The effect of the work for the year is that, in addition to improvements in operating theatres or rooms, nurses' accommodation, sanitary and other requirements, 45 more disused beds will be re-opened and maintained; making a total of 287 re-opened in the last three years through the help of this Fund. Rather more still remain closed, but in many of these instances the beds could not easily be re-opened at the present time. In some hospitals the vacant wards are used for pay patients, and thus fulfil a useful purpose, which makes it desirable to defer the consideration of re-opening these beds as free. In others they are used for nurses, and if they are devoted to their intended purposes fresh accommodation must be provided for nurses. In still harder cases the debts of the hospitals must be liquidated before any fresh liabilities are incurred. In the course of the present Session a petition will be presented to Parliament by the Central Hospital Council, calling attention to the heavy rating of hospitals. In support of this, on behalf of the Fund, a petition has been forwarded in accordance with the instructions of the Executive Committee, expressing the sympathy of all concerned, and their desire to see the rating of all recognised London hospitals abolished.

### Future Work for the Funds.

It may now be considered that the expenses of the Fund have been reduced to a minimum. Every care is taken that the money subscribed be wisely spent. Therefore the General Council would earnestly request all interested in the cause to support the Fund in this present year, when the hospitals will be called upon to admit the wounded, and when more than ever public charity is apt to turn from the sufferers at home to those abroad. Demands must shortly be made on the hospitals not only for their increasing number of patients, but especially this year for soldiers and sailors returning from South Africa in need of good nursing and tender care.

In conclusion, the Council confidently appeal to all their generous supporters, including the Press, to aid them in maintaining the established and successful position of the Fund.

The adoption of the report was moved by the President, the Prince of Wales, seconded by the Bishop of London, and carried unanimously.

Mr. Craggs (in the absence of Lord Rothschild) presented the account of receipts and expenditure and the balance-sheet for the year ending December 31st, 1899, from which it appeared that the receipts were £48,536 15s. 4d., and the total sum distributed £42,000, leaving a balance, after allowing for expenses, of £4,683 2s. 4d. The assets were £176,044 0s. 11d., and the liabilities £24 10s. 4d., leaving a balance of £176,015 12s. 7d.

On the motion of the Prince of WALES (president), seconded by the Duke of FIFE, the account and balance-sheet were adopted and passed.

Mr. SMITH proposed, and Mr. SANDEMAN seconded, that Mr. J. P. Morgan and Mr. W. T. Fry be added to the General Council.

The PRESIDENT: I am sure these two gentlemen will be of valuable assistance to us, as they have already done good work on various committees.

The resolution was then put and carried unanimously.

The PRESIDENT then said: It only remains for me now to perform what may seem a formal duty, but which to me really is one of pleasure, namely, most heartily and sincerely to express my thanks to the General Council, the honorary treasurer, the honorary secretaries, and the honorary auditors for the valuable services that they have rendered to this Fund during the last year. I sincerely hope it will be convenient to them to retain the positions which they now occupy. (Hear, hear.) Many of these gentlemen, I know, have a good many occupations, and they cannot, without inconvenience, devote as much of their time as they would like to this Fund. I may say that one of the members of our Council, the President of the Royal College of Surgeons, Sir William MacCormac, is, as you are all aware, now at the seat of war. At his time of life the performance of the arduous duties that this necessitates reflects, I think, great credit on him, and gives the utmost satisfaction to all who know him. (Hear, hear.) I do not think there is more business to be transacted, unless anybody wishes to bring any other matter before the meeting.

Lord LISTER proposed a vote of thanks to His Royal Highness for the kind, gracious, and effective way he had presided over the meeting.

Lord ROWTON, in seconding the vote, said: May I have the honour to second that resolution, and I beg to repeat the remarks you made, Sir, that I do not think it a mere formality, because I feel confident that the success of this Fund is largely due to the interest your Royal Highness takes in it, and the advice you have given.

The PRESIDENT, in reply, said: I am obliged to you for your kind words. As long as I am so ably supported as now, by these gentlemen around me, and those who are unfortunately unable to be present to-day, I have no doubt the Fund will continue to flourish and be a success in every sense of the word.

The proceedings then terminated.

